# Complete Blood Count-Derived Inflammatory Indices in Prediabetes: A Head-to-Head Comparison

**DOI:** 10.3390/jcm15083160

**Published:** 2026-04-21

**Authors:** Kemal Ozan Lule, Ozge Ozsoy, Omer Yildirim, Hamit Yildiz

**Affiliations:** Department of Internal Medicine, Faculty of Medicine, Gaziantep University, 27410 Gaziantep, Türkiye; ozsoyozgee@gmail.com (O.O.); yldrmomer40@gmail.com (O.Y.); drhyildiz@hotmail.com (H.Y.)

**Keywords:** prediabetic state, inflammation, biomarkers, neutrophils, lymphocytes

## Abstract

**Background:** Chronic low-grade inflammation contributes to early glucose dysregulation, but comparative evidence on complete blood count-derived inflammatory indices in prediabetes remains limited. This study aimed to compare the associations of five complete blood count-derived inflammatory indices with prediabetes and to assess their discriminative performance. **Methods:** In this retrospective cross-sectional study, 255 adults (128 with prediabetes and 127 with normoglycemia) were identified from 12,540 individuals screened at the internal medicine outpatient clinics of a university hospital. The neutrophil-to-lymphocyte ratio, platelet-to-lymphocyte ratio, monocyte-to-lymphocyte ratio, systemic immune-inflammation index, and systemic inflammation response index were compared between groups. Adjusted logistic regression, hierarchical regression, and receiver operating characteristic curve analyses were performed. **Results:** All indices except the monocyte-to-lymphocyte ratio were significantly higher in the prediabetes group (all *p* < 0.001). Among the evaluated indices, the neutrophil-to-lymphocyte ratio showed the strongest association with prediabetes, with the largest standardized odds ratio (2.691; 95% confidence interval, 1.839–3.938) and the highest explanatory power (Nagelkerke R^2^ = 0.326). Its addition to the base model significantly improved model fit (likelihood ratio χ^2^ = 33.62, *p* < 0.001), and the association remained significant after adjustment for body mass index, C-reactive protein, and lipid parameters. It also yielded the highest area under the curve (0.714). **Conclusions:** In this cross-sectional analysis, the neutrophil-to-lymphocyte ratio showed the most robust independent association with prediabetes among the evaluated complete blood count-derived inflammatory indices. However, the overall discriminative performance was modest, supporting the use of these indices as adjunctive rather than standalone screening markers.

## 1. Introduction

Prediabetes is an intermediate metabolic state characterized by glycemic levels above normal but below the diagnostic threshold for diabetes mellitus, including impaired fasting glucose, impaired glucose tolerance, and elevated glycated hemoglobin (HbA1c) [1]. It is associated with an increased risk of progression to type 2 diabetes, with annual conversion rates of 5–10%, and has also been linked to higher cardiovascular morbidity and all-cause mortality [2,3]. Because this stage may still be reversible, the early identification of individuals at increased metabolic risk remains an important clinical priority.

Subclinical inflammatory processes are increasingly implicated in the pathogenesis of insulin resistance and progressive β-cell impairment [4,5]. Evidence suggests that subclinical inflammatory alterations may already be present in prediabetes [6], but their extent and clinical relevance in this early stage of glucose dysregulation have not been fully clarified.

In recent years, inflammatory indices calculated from routine complete blood count parameters have gained considerable attention owing to their low cost, broad accessibility, and ease of computation. The neutrophil-to-lymphocyte ratio (NLR), platelet-to-lymphocyte ratio (PLR), monocyte-to-lymphocyte ratio (MLR), systemic immune-inflammation index (SII), and systemic inflammation response index (SIRI) have been investigated in various cardiometabolic disorders. Although a growing number of reports have examined selected indices such as NLR or SII in relation to glycemic status, these investigations have generally evaluated one or two markers at a time, limiting the ability to determine which index carries the strongest association with early glucose dysregulation [7,8]. Among these, NLR has been the most extensively studied, and a recent meta-analysis showed higher NLR values in individuals with metabolic syndrome than in healthy controls [7]. These indices are thought to integrate signals from both innate and adaptive immunity, potentially capturing dimensions of metabolic inflammation that conventional biomarkers such as CRP may not fully detect [5,9].

However, the majority of prior investigations have examined individual markers in isolation or within populations with established diabetes or metabolic syndrome, with comparatively little attention directed toward the prediabetic stage [8]. As a result, direct comparative evidence across multiple complete blood count-derived inflammatory indices within the same prediabetic population remains limited. A head-to-head evaluation of these indices may therefore help clarify which markers show the most consistent association with early glucose dysregulation and whether their discriminative performance is clinically meaningful.

Accordingly, this study aimed to evaluate the associations of five complete blood count-derived inflammatory indices with prediabetes, compare their relative contributions using adjusted and standardized effect estimates, and assess their discriminative performance.

## 2. Materials and Methods

### 2.1. Study Design and Setting

This retrospective cross-sectional study used hospital information system data from adults attending the Internal Medicine outpatient clinics of Gaziantep University Sahinbey Research and Practice Hospital between 2021 and 2024. The study was conducted in accordance with the Declaration of Helsinki and was approved by the Gaziantep University Non-Interventional Clinical Research Ethics Committee (approval number: 2025/217; approval date: 6 August 2025). Institutional permission for the use of retrospective data was also obtained from the hospital administration.

### 2.2. Participants

**Figure 1:**
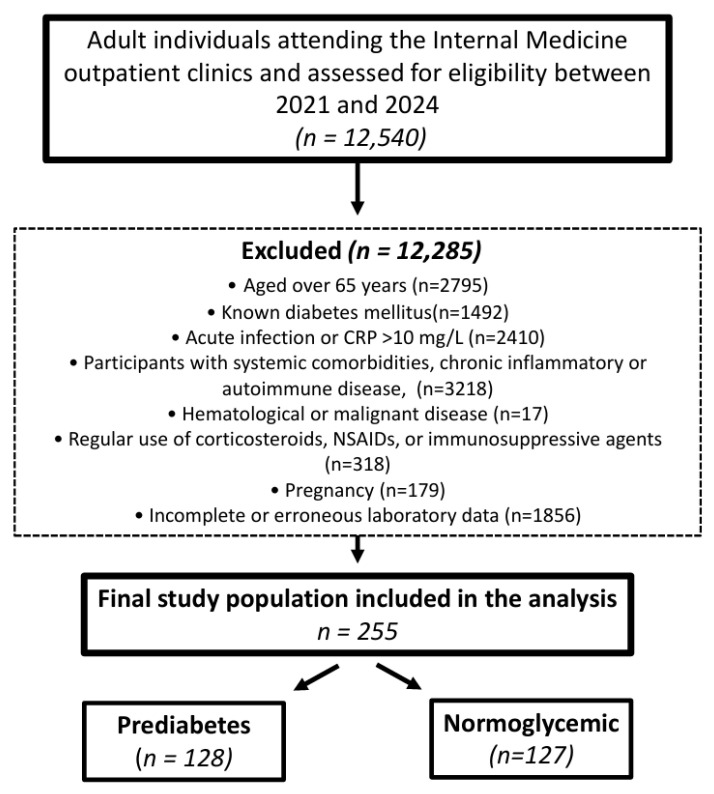
Participant flow diagram.

Adults aged 18–65 years with complete laboratory data available in the hospital information system were eligible for inclusion. Of 12,540 individuals assessed, 255 met the eligibility criteria and were included in the final analysis, comprising 128 participants with prediabetes and 127 normoglycemic controls. The specific reasons for exclusion and the number of individuals excluded at each stage are detailed in the participant flow diagram (Figure 1). Because the exclusion criteria were applied uniformly to all screened individuals, the selection process was systematic and criterion-based rather than discretionary. Nevertheless, the stringent exclusion criteria may have resulted in a healthier analytic sample, and possible selection bias cannot be entirely ruled out.

Prediabetes was defined according to the American Diabetes Association criteria [1] as fasting plasma glucose levels of 100–125 mg/dL and/or glycated hemoglobin (HbA1c) levels of 5.7–6.4%. Normoglycemia was defined as fasting plasma glucose <100 mg/dL and HbA1c < 5.7%. Because this was a retrospective study relying on routinely recorded data, oral glucose tolerance testing results were not systematically available in the hospital information system; consequently, individuals with isolated impaired glucose tolerance may not have been identified.

Participants were excluded if they had diabetes mellitus, evidence of acute infection, C-reactive protein (CRP) levels > 10 mg/L [10], chronic inflammatory or autoimmune disease, hematological disease, malignancy, pregnancy, regular use of corticosteroids, non-steroidal anti-inflammatory drugs, or immunosuppressive agents, or incomplete or erroneous laboratory records.

Flow diagram of participant selection from 12,540 adults screened in the Internal Medicine outpatient clinics of Gaziantep University Sahinbey Research and Practice Hospital between 2021 and 2024, resulting in the inclusion of 255 participants in the final analysis.

### 2.3. Laboratory Measurements and Derived Inflammatory Indices

Laboratory parameters were obtained from routine outpatient evaluations recorded in the hospital information system. Complete blood count parameters were analyzed using a Sysmex XN-9000 hematology analyzer (Sysmex Corporation, Kobe, Japan), HbA1c was measured using a Lifotronic H9 glycated hemoglobin analyzer (Lifotronic, Shenzhen, China), and biochemical parameters were analyzed using a Beckman Coulter AU5800 clinical chemistry analyzer (Beckman Coulter, Inc., Brea, CA, USA). All measurements were performed in the central laboratory of the hospital, which participates in an external quality assessment programme and applies daily internal quality control procedures in accordance with the manufacturer’s specifications. The analyzed variables included fasting plasma glucose, HbA1c, CRP, platelet count, triglycerides (TG), high-density lipoprotein cholesterol (HDL-C), low-density lipoprotein cholesterol (LDL-C), creatinine, and complete blood count parameters.

Complete blood count-derived inflammatory indices were calculated from absolute cell counts obtained from the same blood sample as follows: neutrophil-to-lymphocyte ratio (NLR) = neutrophil/lymphocyte; platelet-to-lymphocyte ratio (PLR) = platelet/lymphocyte; monocyte-to-lymphocyte ratio (MLR) = monocyte/lymphocyte; systemic immune-inflammation index (SII) = platelet × neutrophil/lymphocyte; and systemic inflammation response index (SIRI) = neutrophil × monocyte/lymphocyte. To improve interpretability in regression analyses, SII was entered per 100-unit increase and PLR per 10-unit increase.

### 2.4. Outcome Measures

The primary outcome was the association between complete blood count-derived inflammatory indices and prediabetes. Secondary outcomes were the comparative discriminative performance of these indices and the identification of an optimal NLR cut-off value.

### 2.5. Sample Size Estimation

Sample size estimation was performed using G*Power version 3.9.1 and indicated a minimum of 104 participants per group (total *n* = 208), based on Cohen’s d = 0.39 [11], an α level of 0.05, and 80% power. The final sample of 255 exceeded this requirement.

### 2.6. Statistical Analysis

Statistical analyses were performed using IBM SPSS Statistics version 22 (IBM Corp., Armonk, NY, USA). Normality was assessed visually and analytically. Continuous variables are presented as mean ± standard deviation or median (25th–75th percentile), as appropriate, and categorical variables as number (%).

Between-group comparisons were performed using the independent-samples *t*-test, Mann–Whitney U test, chi-square test, or Fisher’s exact test, as appropriate. Correlations between inflammatory indices and metabolic parameters were assessed using Spearman’s rank correlation analysis.

Binary logistic regression was used to evaluate the independent association of each inflammatory index with prediabetes in separate models adjusted for age, sex, and body mass index (BMI). Because the five inflammatory indices are expressed on different numerical scales (e.g., NLR typically ranges from 1 to 5, whereas SII may range from 200 to 1500), direct comparison of their unstandardized regression coefficients would be misleading. To address this, each index was transformed to z-scores (mean = 0, SD = 1) prior to entry into the regression models, so that the resulting per-SD odds ratios reflect a comparable unit of change across all indices and enable meaningful head-to-head comparison of effect sizes.

A hierarchical logistic regression model was subsequently constructed to assess the incremental contribution of NLR beyond conventional risk factors. Age, sex, and BMI were entered in the first block, followed by NLR, CRP, and lipid parameters (TG and HDL-C) in subsequent blocks. Incremental model contribution was evaluated using likelihood ratio testing, and model calibration was assessed with the Hosmer–Lemeshow test. Multicollinearity among independent variables was assessed using variance inflation factors derived from a linear regression model containing the same covariates, with a threshold of 5 indicating potentially problematic multicollinearity.

Receiver operating characteristic (ROC) curve analysis was performed to evaluate the discriminative performance of the inflammatory indices. The area under the curve (AUC) with 95% confidence intervals was calculated. Pairwise comparisons of AUC values were performed using the DeLong test in MedCalc Statistical Software version 23 (MedCalc Software Ltd., Ostend, Belgium). The optimal NLR cut-off value was determined using the Youden index and was subsequently used in categorical analyses. Because each inflammatory index was evaluated in a separate regression model rather than simultaneously in a single model, formal adjustment for multiple comparisons (e.g., Bonferroni correction) was not applied. This approach was chosen because the primary aim was not to test a family of independent hypotheses but to compare the relative strength of association of each index with prediabetes within a consistent analytic framework. Nevertheless, the results should be interpreted with this consideration in mind. All tests were two-sided, and *p* < 0.05 was considered statistically significant.

## 3. Results

### 3.1. Baseline Characteristics of the Study Population

A total of 255 participants were included, comprising 127 normoglycemic individuals and 128 with prediabetes. Baseline characteristics are presented in Table 1. The prediabetes group had a higher proportion of women (56.3% vs. 41.7%, *p* = 0.020) and higher BMI (29.82 ± 4.66 vs. 26.81 ± 3.51 kg/m^2^, *p* < 0.001), while age was comparable between groups (*p* = 0.071). As expected, fasting glucose and HbA1c were significantly higher in the prediabetes group (both *p* < 0.001). Among inflammatory markers, CRP (median 5.10 vs. 2.90 mg/L), NLR (2.64 vs. 1.80), PLR (146.56 vs. 113.82), SII (694.22 vs. 490.64), and SIRI (1.51 vs. 1.08) were all significantly elevated in the prediabetes group (all *p* < 0.001), whereas MLR did not differ between groups (*p* = 0.851). Triglycerides were higher (*p* < 0.001) and HDL-C lower (*p* = 0.014) in the prediabetes group, while LDL-C, platelet count, and creatinine were similar.

### 3.2. Correlation Analyses

Spearman correlation analysis between inflammatory indices and metabolic parameters is presented in Table 2. Among the evaluated indices, the neutrophil-to-lymphocyte ratio (NLR) showed the strongest correlation with HbA1c (rho = 0.46, *p* < 0.01) and was also positively correlated with C-reactive protein (CRP) (rho = 0.42, *p* < 0.01) and, more weakly, with glucose (rho = 0.15, *p* < 0.05). The platelet-to-lymphocyte ratio (PLR), systemic immune-inflammation index (SII), and systemic inflammation response index (SIRI) were also positively correlated with HbA1c and CRP, although these correlations were generally weaker than those observed for NLR. PLR was additionally positively correlated with body mass index (BMI) (rho = 0.21, *p* < 0.01), whereas SII showed a weak positive correlation with glucose (rho = 0.13, *p* < 0.05). In contrast, the monocyte-to-lymphocyte ratio (MLR) was not significantly correlated with BMI, glucose, HbA1c, CRP, or HDL-C, and showed only a weak negative correlation with triglycerides (TG) (rho = −0.14, *p* < 0.05). Overall, HbA1c and CRP showed the most consistent positive correlations with the evaluated inflammatory indices.

### 3.3. Regression Analyses

Age-, sex-, and body mass index (BMI)-adjusted logistic regression analyses showed that the neutrophil-to-lymphocyte ratio (NLR), systemic inflammation response index (SIRI), systemic immune-inflammation index (SII), and platelet-to-lymphocyte ratio (PLR) were all significantly associated with prediabetes (Table 3). Among these indices, NLR showed the strongest association, with the highest adjusted odds ratio on the original scale (OR = 2.553, 95% CI: 1.781–3.661, *p* < 0.001) and the largest standardized effect size (per-SD OR = 2.691, 95% CI: 1.839–3.938, *p* < 0.001). NLR also showed the greatest explanatory power among the evaluated models (Nagelkerke R^2^ = 0.326). Although SIRI, SII, and PLR were also significantly associated with prediabetes, their standardized effect sizes and explanatory power were lower than those of NLR.

### 3.4. Hierarchical Logistic Regression

Because NLR showed the strongest association in the adjusted regression analyses, it was selected for hierarchical logistic regression modelling (Table 4). In the base model including age, sex, and BMI, only BMI was independently associated with prediabetes. The addition of NLR in Model 2 significantly improved model fit (LRT χ^2^ = 33.62, *p* < 0.001), increasing Nagelkerke R^2^ from 0.184 to 0.326, and NLR remained independently associated with prediabetes. Further inclusion of CRP in Model 3 provided an additional improvement in model fit (LRT χ^2^ = 10.77, *p* = 0.001), with both NLR and CRP remaining significant. In the fully adjusted model, the inclusion of TG and HDL-C further improved model fit (LRT χ^2^ = 9.73, *p* = 0.008), and NLR, CRP, BMI, and TG remained independently associated with prediabetes, whereas HDL-C did not. The final model had the highest explanatory power (Nagelkerke R^2^ = 0.404) and showed adequate calibration according to the Hosmer–Lemeshow test.

### 3.5. Discriminative Performance

**Figure 2:**
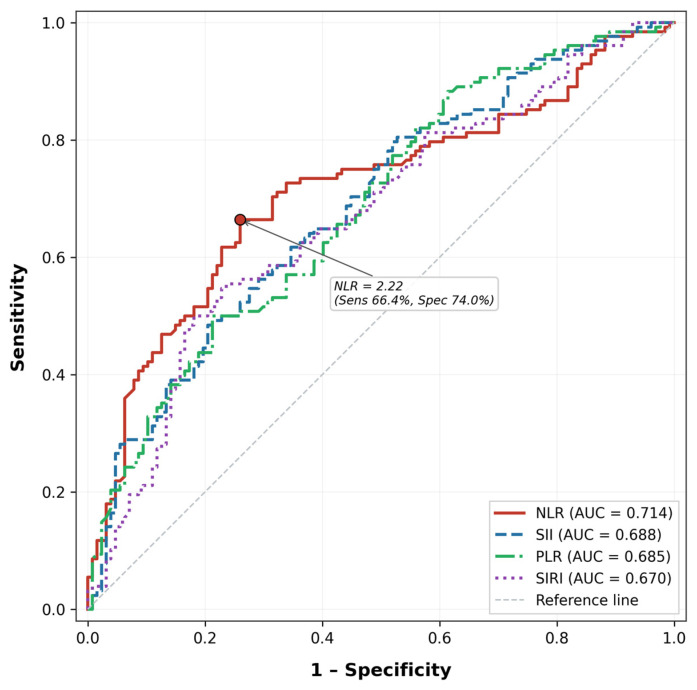
Receiver operating characteristic curves of hematological inflammatory indices for identifying prediabetes. Receiver operating characteristic curves for NLR, PLR, MLR, SII, and SIRI. NLR showed the highest area under the curve among the evaluated indices. NLR, neutrophil-to-lymphocyte ratio; PLR, platelet-to-lymphocyte ratio; MLR, monocyte-to-lymphocyte ratio; SII, systemic immune-inflammation index; SIRI, systemic inflammation response index.

ROC analyses are presented in Figure 2. NLR had the highest AUC among the evaluated inflammatory indices (0.714; 95% CI, 0.650–0.778; *p* < 0.001), although its discriminative performance remained modest. In pairwise DeLong comparisons, the AUC of NLR was significantly higher only than that of SIRI. The optimal NLR cut-off value was 2.22, and participants with NLR values at or above this threshold had a higher frequency of prediabetes. In additional logistic regression analysis using this ROC-derived threshold, elevated NLR remained independently associated with prediabetes after adjustment for age, sex, and BMI (adjusted OR, 5.52; 95% CI, 3.06–10.00; *p* < 0.001).

## 4. Discussion

In this retrospective cross-sectional study, NLR, PLR, SII, and SIRI were higher in individuals with prediabetes, whereas MLR did not differ significantly between groups. Among the evaluated complete blood count-derived inflammatory indices, NLR showed the strongest independent association with prediabetes in standardized analyses. Although NLR also yielded the highest discriminative performance, the overall AUC values were modest, indicating that these indices may be more useful as complementary rather than standalone tools for early metabolic risk assessment.

The observation of higher NLR values among individuals with prediabetes aligns with a growing body of evidence implicating this ratio in early glycemic disturbance and adverse cardiometabolic profiles. Population-based studies have shown that higher NLR is associated with adverse outcomes in individuals with prediabetes or diabetes, and meta-analytic evidence has demonstrated higher NLR values in metabolic syndrome than in healthy controls [7,12,13]. The present results build upon this evidence by demonstrating that the NLR–prediabetes association persisted following sequential adjustment for adiposity, acute-phase inflammation, and lipid profile, suggesting that NLR may capture inflammatory variation not fully explained by adiposity or conventional inflammatory markers. The relevance of evaluating these indices specifically in prediabetes lies in the fact that this stage represents an earlier and potentially reversible phase of metabolic dysfunction. In contrast to overt diabetes, where inflammatory and metabolic disturbances are already more established, the identification of accessible inflammatory signals in prediabetes may be more informative for early risk stratification and preventive assessment.

A possible explanation for the comparatively stronger performance of NLR is that it integrates two complementary components of metabolic inflammation: neutrophil-driven innate immune activation and relative lymphocyte suppression [4,5,9,14,15]. Previous studies have also linked NLR to insulin resistance across the spectrum of glucose dysregulation [16,17]. Although insulin resistance indices were not available in the present study, the persistence of the NLR association after adjustment for BMI, CRP, and lipid parameters supports the view that NLR may be associated with early metabolic inflammatory burden beyond conventional risk markers. This pattern may also explain why NLR performed more consistently than indices with greater structural complexity, which do not necessarily provide additional discriminatory value in the setting of early dysglycemia.

Importantly, the independent association of NLR with prediabetes persisted after sequential adjustment for CRP in the hierarchical regression model. This finding suggests that NLR captures an inflammatory dimension not fully reflected by CRP alone. While CRP is primarily a hepatic acute-phase reactant driven by interleukin-6 signalling [10], NLR reflects the balance between innate immune effector cells and adaptive regulatory components, thereby providing complementary information about the immunological milieu [14]. The retained significance of both NLR and CRP in the fully adjusted model is consistent with the possibility that these two markers capture partially non-overlapping aspects of the inflammatory response observed in early glucose dysregulation.

SII, SIRI, and PLR were also independently associated with prediabetes, although their standardized effect sizes and explanatory power were lower than those of NLR. Recent studies have reported associations between higher SII and prediabetes, insulin resistance, and glycemic markers [18,19], and Tuzimek et al. showed that SII, SIRI, and related indices were associated with acute coronary syndrome risk in patients with diabetes or prediabetes [20]. Within the present comparative framework, however, these more complex composite indices did not outperform NLR. This finding suggests that greater index complexity does not necessarily translate into a stronger association with early glucose dysregulation.

From a methodological standpoint, it is noteworthy that the simplest index evaluated—NLR, derived from only two cell lineages—consistently outperformed the more complex composite indices such as SII and SIRI, which incorporate three or more cell types. This observation aligns with the principle of parsimony and has practical implications: NLR can be calculated with minimal effort from any standard complete blood count report, without requiring additional parameters or computational steps. In the context of prediabetes screening in resource-limited or primary care settings, a simpler marker with comparable or superior performance may be more readily adoptable than composite indices [7,8].

By contrast, MLR was not associated with prediabetes in our cohort. Monocytes play an established role in adipose tissue inflammation and macrophage recruitment in obesity and overt diabetes [4], but monocyte-related alterations may be too subtle to distinguish individuals at the prediabetic stage. This interpretation is consistent with previous observations suggesting that leukocyte-derived indices may show differential sensitivity across the spectrum from prediabetes to overt diabetes [17].

From a clinical perspective, the relevance of these findings lies not in proposing NLR as a standalone diagnostic test for prediabetes, but in highlighting its potential value as an accessible adjunctive marker within routine metabolic risk assessment. Because NLR can be derived from a standard complete blood count without additional cost or testing burden, it may help identify individuals with prediabetes who exhibit a more pronounced inflammatory-metabolic profile. This may be particularly relevant in settings where more comprehensive metabolic or inflammatory assessment is not readily available [7,8,14]. Indeed, given the modest discriminative accuracy of NLR alone (AUC = 0.714), its clinical value is unlikely to lie in isolated use but rather in combination with established metabolic risk factors. The hierarchical regression analysis in the present study showed that model performance improved incrementally when NLR was combined with BMI, CRP, and lipid parameters (final model Nagelkerke R^2^ = 0.404), suggesting that integrating NLR into composite risk models may yield more clinically meaningful stratification than any single marker in isolation.

Nevertheless, the discriminative performance of NLR was modest and, in pairwise comparisons, not clearly superior to that of several other indices. Accordingly, these markers should not be interpreted as standalone diagnostic tools for prediabetes. Rather, they may be more appropriately viewed as pragmatic adjunctive markers that could contribute to broader risk assessment frameworks.

The optimal NLR cut-off of 2.22 identified in this study is broadly consistent with thresholds reported in related clinical contexts. Using NHANES data, Dong et al. identified an NLR cut-off of 2.14 as the optimal threshold for predicting all-cause mortality in individuals with prediabetes [12]. In a study of diabetic nephropathy, Subramani et al. reported a similar NLR threshold of 2.2 with 72.3% sensitivity and 78.1% specificity [21]. Although the convergence of these values across different endpoints and populations is noteworthy, this apparent consistency should be interpreted with caution, as the compared studies differed in design, population characteristics, and clinical outcomes. The observed similarity in thresholds does not constitute evidence of reproducibility. However, the cut-off in the present study was derived from the same dataset used for the primary analysis, which introduces the risk of overfitting. This threshold should therefore be regarded as exploratory, and its clinical applicability remains uncertain until confirmed through external validation in independent and ethnically diverse cohorts.

### 4.1. Limitations

This study has several limitations that merit consideration. First, the cross-sectional retrospective design does not permit causal conclusions or determination of the temporal sequence between inflammatory marker elevation and glycemic deterioration. Second, despite the application of strict exclusion criteria, unmeasured confounders—including dietary patterns, habitual physical activity levels, and regional fat distribution—may have influenced the observed associations. Third, the NLR cut-off of 2.22 was derived from the same dataset and was not externally validated. Fourth, insulin measurements were unavailable, precluding direct assessment of insulin resistance indices such as HOMA-IR. As insulin resistance represents a central pathophysiological link between systemic inflammation and glucose dysregulation, the absence of these measurements limits the ability to determine whether the observed associations between inflammatory indices and prediabetes are mediated through insulin resistance or through alternative metabolic pathways. Future studies incorporating fasting insulin and HOMA-IR would help clarify the biological mechanisms underlying these associations. Finally, formal correction for multiple comparisons was not applied, and the possibility of inflated type I error cannot be excluded.

Furthermore, prediabetes was defined using fasting plasma glucose and HbA1c without oral glucose tolerance testing, which may have resulted in misclassification of individuals with isolated impaired glucose tolerance. Such misclassification would most likely result in the inclusion of some individuals with undetected impaired glucose tolerance in the normoglycemic control group, which could attenuate the observed between-group differences and lead to conservative effect estimates. Additionally, the study population was drawn from a single university hospital in southeastern Türkiye, a region with distinct dietary habits, genetic background, and metabolic risk profiles. These population-specific characteristics may limit the generalizability of both the observed associations and the proposed NLR cut-off value to other ethnic, geographic, or healthcare settings. Multicentre studies involving diverse populations are warranted to establish whether these findings are reproducible across different demographic contexts.

### 4.2. Strengths

This study has several strengths. First, the large initial screening population allowed stringent exclusion criteria to be applied, resulting in a well-characterized analytic cohort. Second, multiple complete blood count-derived inflammatory indices were evaluated simultaneously within the same study population, enabling direct comparison across markers. Third, the use of both original-scale and standardized effect estimates, together with hierarchical regression modelling and pairwise AUC comparisons, strengthened the robustness of the comparative analytic framework.

## 5. Conclusions

Among the evaluated complete blood count-derived inflammatory indices, NLR showed the strongest and most consistent independent association with prediabetes. However, the overall discriminative performance of these markers was modest, indicating that they should not be considered standalone screening tools. Their potential clinical value may lie in supporting routine metabolic risk stratification as simple and readily available adjunctive inflammatory markers. Prospective multicenter studies incorporating insulin resistance measures and external validation cohorts are needed to clarify their role in early metabolic risk assessment.

## Figures and Tables

**Table 1:** Baseline characteristics of the study population by glycemic status.

Variable	Normoglycemic (*n* = 127)	Prediabetes (*n* = 128)	*p*
Sex, *n* (%)			0.020
Female	53 (41.7)	72 (56.3)	
Male	74 (58.3)	56 (43.8)	
Age, years	44.8 (12.6)	47.6 (11.4)	0.071
BMI, kg/m^2^	26.81 (3.51)	29.82 (4.66)	<0.001
Fasting glucose, mg/dL	88.0 (80.0–91.0)	103.0 (90.3–112.0)	<0.001
HbA1c, %	5.05 (0.37)	5.80 (0.39)	<0.001
CRP, mg/L	2.90 (1.20–5.00)	5.10 (2.53–7.00)	<0.001
Platelet count, ×10^9^/L	280.6 (74.2)	280.9 (64.8)	0.970
NLR	1.80 (1.48–2.29)	2.64 (1.89–3.36)	<0.001
PLR	113.82 (91.17–145.00)	146.56 (113.28–225.50)	<0.001
MLR	0.23 (0.18–0.30)	0.24 (0.18–0.30)	0.851
SII	490.64 (361.85–691.88)	694.22 (494.48–987.60)	<0.001
SIRI	1.08 (0.76–1.37)	1.51 (1.04–2.07)	<0.001
TG, mg/dL	118.0 (93.0–173.0)	148.0 (122.0–222.5)	<0.001
HDL-C, mg/dL	47.5 (12.0)	43.8 (11.9)	0.014
LDL-C, mg/dL	129.9 (29.9)	135.3 (31.9)	0.171
Creatinine, mg/dL	0.74 (0.18)	0.75 (0.17)	0.812

Normally distributed continuous variables are presented as mean (SD), and non-normally distributed continuous variables as median (25th–75th percentile). Categorical variables are presented as *n* (%). Between-group comparisons were performed using the independent samples *t*-test, Mann–Whitney U test, chi-square test, or Fisher’s exact test, as appropriate. BMI, body mass index; HbA1c, glycated hemoglobin; CRP, C-reactive protein; NLR, neutrophil-to-lymphocyte ratio; PLR, platelet-to-lymphocyte ratio; MLR, monocyte-to-lymphocyte ratio; SII, systemic immune-inflammation index; SIRI, systemic inflammation response index; TG, triglycerides; HDL-C, high-density lipoprotein cholesterol; LDL-C, low-density lipoprotein cholesterol.

**Table 2:** Spearman correlation analysis between inflammatory indices and metabolic parameters (*n* = 255).

Variable	BMI	Glucose	HbA1c	CRP	TG	HDL-C
NLR	0.08	0.15 *	0.46 **	0.42 **	0.00	−0.08
PLR	0.21 **	0.09	0.39 **	0.27 **	0.08	−0.03
MLR	−0.08	−0.11	0.02	0.04	−0.14 *	−0.05
SII	0.10	0.13 *	0.39 **	0.42 **	0.07	−0.02
SIRI	0.02	0.04	0.38 **	0.34 **	−0.01	−0.07

Spearman correlation coefficients (rho) are presented. * *p* < 0.05, ** *p* < 0.01. BMI, body mass index; HbA1c, glycated hemoglobin; CRP, C-reactive protein; TG, triglycerides; HDL-C, high-density lipoprotein cholesterol; NLR, neutrophil-to-lymphocyte ratio; PLR, platelet-to-lymphocyte ratio; MLR, monocyte-to-lymphocyte ratio; SII, systemic immune-inflammation index; SIRI, systemic inflammation response index.

**Table 3:** Age-, sex-, and BMI-adjusted logistic regression analyses of inflammatory indices associated with prediabetes: original-scale and standardized effect sizes.

Index	Adjusted OR (95% CI)	*p*	Per-SD OR (95% CI)	*p*	Nagelkerke R^2^
NLR	2.553 (1.781–3.661)	<0.001	2.691 (1.839–3.938)	<0.001	0.326
SIRI	2.129 (1.462–3.098)	<0.001	2.028 (1.427–2.882)	<0.001	0.267
SII per 100-unit increase	1.226 (1.110–1.354)	<0.001	1.995 (1.426–2.792)	<0.001	0.267
PLR per 10-unit increase	1.085 (1.038–1.134)	<0.001	1.823 (1.315–2.526)	<0.001	0.248

Each inflammatory index was entered into a separate logistic regression model adjusted for age, sex, and BMI. Adjusted ORs are presented on the original scale of each index (NLR and SIRI per unit increase; SII per 100-unit increase; PLR per 10-unit increase). Per-SD ORs represent the odds ratio associated with a one-standard-deviation increase after z-score transformation. Nagelkerke R^2^ values are derived from the standardized models. OR, odds ratio; CI, confidence interval; SD, standard deviation; NLR, neutrophil-to-lymphocyte ratio; SIRI, systemic inflammation response index; SII, systemic immune-inflammation index; PLR, platelet-to-lymphocyte ratio; BMI, body mass index.

**Table 4:** Hierarchical logistic regression analysis of factors associated with prediabetes.

Variable	Model 1 OR (95% CI)	*p*	Model 2 OR (95% CI)	*p*	Model 3 OR (95% CI)	*p*	Model 4 OR (95% CI)	*p*
Age	1.018 (0.996–1.041)	0.111	1.014 (0.990–1.038)	0.254	1.015 (0.991–1.040)	0.219	1.014 (0.989–1.040)	0.277
Sex	0.607 (0.357–1.030)	0.064	0.848 (0.476–1.512)	0.577	0.979 (0.539–1.777)	0.944	0.849 (0.458–1.572)	0.602
BMI	1.187 (1.110–1.269)	<0.001	1.201 (1.117–1.292)	<0.001	1.213 (1.123–1.309)	<0.001	1.205 (1.115–1.303)	<0.001
NLR	—	—	2.553 (1.781–3.661)	<0.001	2.163 (1.490–3.139)	<0.001	2.160 (1.468–3.177)	<0.001
CRP	—	—	—	—	1.229 (1.084–1.392)	0.001	1.223 (1.077–1.390)	0.002
TG	—	—	—	—	—	—	1.004 (1.001–1.008)	0.018
HDL-C	—	—	—	—	—	—	0.980 (0.956–1.005)	0.124
−2 Log likelihood	315.583		281.965		271.193		261.465	
Nagelkerke R^2^	0.184		0.326		0.368		0.404	
LRT χ^2^ (df)	—		33.62 (1)		10.77 (1)		9.73 (2)	
LRT *p*	—		<0.001		0.001		0.008	
H-L *p*	0.067		0.688		0.726		0.850	

Binary logistic regression with hierarchical entry was performed. Model 1 included age, sex, and BMI; Model 2 included Model 1 plus NLR; Model 3 included Model 2 plus CRP; and Model 4 included Model 3 plus TG and HDL-C. Sex was coded as 0 = female and 1 = male. LRT χ^2^ (df) indicates the likelihood ratio chi-square statistic and its degrees of freedom, where df equals the number of variables added at each step (Model 2: NLR, df = 1; Model 3: CRP, df = 1; Model 4: TG and HDL-C, df = 2). All models showed adequate calibration according to the Hosmer-Lemeshow test (all *p* > 0.05). Overall classification accuracy of the final model was 74.1%. All variance inflation factor values were <1.28. OR, odds ratio; CI, confidence interval; BMI, body mass index; NLR, neutrophil-to-lymphocyte ratio; CRP, C-reactive protein; TG, triglycerides; HDL-C, high-density lipoprotein cholesterol; LRT, likelihood ratio test; H-L, Hosmer-Lemeshow test.

## Data Availability

The datasets analyzed during the current study are not publicly available because of institutional data protection policies but are available from the corresponding author on reasonable request.

## Abbreviations

The following abbreviations are used in this manuscript:

AUC	Area under the curve
BMI	Body mass index
CBC	Complete blood count
CI	Confidence interval
CRP	C-reactive protein
H-L	Hosmer–Lemeshow
HbA1c	Glycated haemoglobin
HDL-C	High-density lipoprotein cholesterol
HOMA-IR	Homeostatic model assessment of insulin resistance
LDL-C	Low-density lipoprotein cholesterol
LRT	Likelihood ratio test
MLR	Monocyte-to-lymphocyte ratio
NLR	Neutrophil-to-lymphocyte ratio
OR	Odds ratio
PLR	Platelet-to-lymphocyte ratio
ROC	Receiver operating characteristic
SD	Standard deviation
SII	Systemic immune-inflammation index
SIRI	Systemic inflammation response index
TG	Triglycerides
